# An Experimental Study of Interfacial Dynamics Control Using Temperature-Sensitive Surfactants

**DOI:** 10.1021/acs.langmuir.5c06748

**Published:** 2026-04-28

**Authors:** Amirhosein Sarchami, Saaras Pakanati, Ayaaz Yasin, Milind A. Jog, Kishan Bellur

**Affiliations:** Department of Mechanical and Materials Engineering, 212544 University of Cincinnati, Cincinnati, Ohio 45220, United States

## Abstract

The dynamic control of meniscus curvature is a fundamental aspect of interfacial science with broad implications for microfluidics, thermal management, and adaptive surface technologies. Here, we investigate the temperature-induced interfacial dynamics of menisci in oil–water systems containing thermoresponsive surfactants. Using high-resolution bright-field microscopy, we systematically examine the effects of temperature-sensitive surfactants, concentration, capillary geometry, temporal temperature gradients, contact angle hysteresis, and heat transfer direction (heating/cooling) on meniscus displacement, contact angle, and interfacial tension. Our results demonstrate that the combination of C_18_TAB in water and hexadecanol in hexadecane enables reversible and tunable switching between concave and convex meniscus shapes, with the transition temperature (*T*_t_) strongly dependent on surfactant composition and concentration. A unique thermal hysteresis in contact angle is observed and is attributed to the asymmetric adsorption/desorption kinetics and interfacial freezing, which is amplified in wider capillaries. However, temporal temperature gradients within the tested range have minimal influence on curvature switching, indicating quasi-static interfacial equilibration at supra-CMC concentrations. These findings establish a framework for actively manipulating interfacial geometry, providing design principles for programmable and reversible wettability control in interfacial systems.

## Graphical abstract

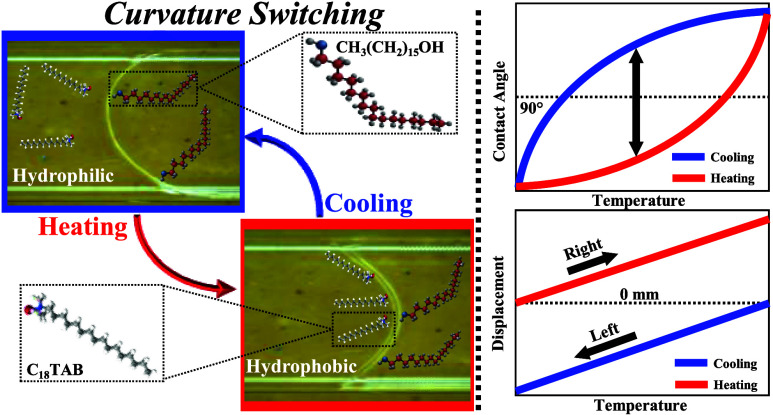

## Introduction

Liquid–liquid and liquid–vapor interfaces are often characterized by a concave or convex meniscus, a key manifestation of wettability and capillary action.^1,2^ A concave meniscus (on hydrophilic surfaces) promotes capillary rise, driving liquid transport in confined geometries. This underlies many natural and engineered processes, including water uptake in plants, fluid infiltration in soils, oil recovery, ink transport, liquid wicking in textiles, brine transport in solar evaporators, and passive fluid management in heat pipes.^3–5^ Conversely, a convex meniscus (on hydrophobic surfaces) expels liquid, enabling technologies like dirt-repellent coatings, self-cleaning fabrics, water-resistant glass, antifogging/antifouling surfaces, droplet shedding for condensation heat transfer, and other surface engineering strategies.^6–9^

Controlling interfacial dynamics, and thereby the wettability and capillary action of the liquid–liquid and/or liquid–vapor meniscus, has been a historical objective in both fundamental research and applied technologies.^10,11^ Such control has primarily relied on passive methods, including modifying the liquid’s chemical composition,^12^ applying permanent surface treatments (e.g., hydrophobic or hydrophilic coatings),^9^ and using fixed surface patterning.^13^ While these static methods have enabled substantial advancements, they inherently suffer from limited flexibility, irreversibility, or complexity. Surface treatments and chemical modifications are typically fixed once applied, constraining real-time alteration and repeatability.

In contrast to passive methods, active interfacial dynamics control refers to the ability to reversibly modulate interfacial properties such as contact angle, position of the interface, and surface tension in response to external stimuli in real time. This strategy offers substantial advantages by enabling spatiotemporal control over fluid interfaces. Active methods allow systems to adapt dynamically to changing conditions or operational demands without relying on permanent surface treatments or elaborate infrastructure.^14–16^ The ability to dynamically control meniscus curvature opens up possibilities for a wide array of technologies, including liquid lenses,^17^ interfacial assembly,^18^ microfluidics,^19^ direct-write three-dimensional (3D) printing of functional materials,^20^ flow in porous media,^21^ thermal management systems,^22–24^ and microelectronic device fabrication.^25,26^ Several approaches have been explored to achieve active interfacial modulation, including the application of electrowetting-on-dielectric,^27,28^ ferrofluid-based wettability tuning,^29^ acoustic fields,^30^ photoresponsive surfactants and coatings,^31^ pH-responsive polymers and surfactants,^7,32^ and thermoresponsive surfactants or polymer brushes.^8,33^ These approaches enable reversible interfacial changes and controlled tuning of interfacial energies, but each comes with limitations. For example, external-field methods typically rely on bulky, energy-intensive equipment.^11^

Almost all active and passive methods can be applied to both liquid–liquid and liquid–vapor interfaces. However, liquid–liquid (oil–water) interfaces exhibit equally rich and complex meniscus behavior, governed primarily by interfacial tension and wettability, unlike liquid–vapor interfaces, where evaporative effects are also significant.^18,34^ In confined geometries such as capillaries, oil–water menisci can undergo spontaneous reconfiguration driven by differential wetting of the channel walls, temperature-induced variations in interfacial tension, or surfactant partitioning between the two fluid phases. These dynamics play a central role in a variety of processes, including emulsion formation and breakup, solvent extraction, enhanced oil recovery, droplet microfluidics, and the interfacial assembly of nanoparticles and surfactant aggregates.^19,35^

Among dynamic strategies, stimuli-responsive materials, particularly temperature-sensitive surfactants, are highly promising due to their simplicity, applicability, and effectiveness. Wang et al.^36^ used a temperature-induced aqueous surfactant (DBAB, DPAB, DEAB) in a two-phase system to show how temperature drives surfactant assemblies to reorganize at interfaces. The transition temperature, at which wettability changes, can be tuned via surfactant composition or small additives, highlighting the roles of hydrophobic interactions and cationic–anionic cooperativity. Feng et al.^34^ developed thermoresponsive surfactants (hexadecanol) with a lower critical solution temperature (LCST) near 34 °C to control emulsion stability. Interfacial tension and morphology measurements confirmed that temperature-dependent surfactant organization governs reversible emulsion stability. Recently, Shool et al.^35^ demonstrated reversible wettability alteration solely via temperature, leveraging thermally activated surfactants. This method enables active, energy-efficient interfacial manipulation without permanent surface modification or continuous external fields, allowing straightforward control of fluid interfaces.

Despite prior demonstrations of temperature-induced changes in wettability, several critical gaps remain. Specifically, the interplay between surfactant concentration, thermal gradients, capillary geometry, and heating/cooling sequences, along with their combined effects on meniscus curvature, transition temperature, and hysteresis are not fully understood. Moreover, the repeatability, stability, and timing of curvature switching under practical thermal conditions are largely unexplored. Addressing these gaps is essential for the development of reliable, programmable interfacial control strategies that can be directly applied in microfluidics, thermal management, adaptive optics, and other technologies requiring dynamic fluid-interface manipulation. In this study, we experimentally investigate surfactant-driven interfacial dynamics across different capillary geometries containing temperature-responsive surfactants (C_18_TAB and CH_3_(CH_2_)_15_OH), combining high-resolution imaging and temperature-controlled experiments. We investigate how the temperature-induced wettability dynamics influence interfacial properties, including contact angle, meniscus displacement, and surface tension, during heating and cooling cycles. High-resolution bright-field microscopy captures and quantifies meniscus evolution under controlled thermal conditions. This work provides fundamental insights into the mechanisms governing reversible, tunable curvature switching, establishing design principles for actively controlling liquid–liquid and liquid–vapor interfaces in real-world applications.

## Theory

Before examining the factors influencing surfactant-driven wettability transitions during heating and cooling, it is essential to understand the underlying mechanisms that enable active control over interfacial properties. As shown in Figure 1, this phenomenon, hereafter referred to as curvature switching, describes a reversible transformation of the meniscus shape from concave to convex, or vice versa, as the temperature varies. This geometric change alters both the direction and magnitude of the capillary pressure, posing challenges for conventional microfluidic systems, thermal management devices, and other interfacial applications that typically assume temperature-independent curvature.

**Figure 1:**
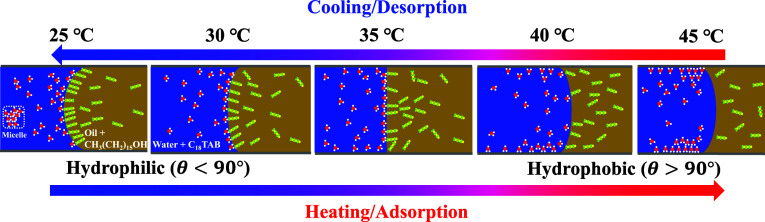
Illustration of the curvature switching phenomenon, where the meniscus transitions between concave and convex states with temperature variation, reversing the direction and magnitude of capillary pressure. This behavior results from two coupled mechanisms: (1) temperature-dependent adsorption/desorption of C_18_TAB surfactant molecules on the aqueous side, which modifies contact angle and surface wettability; and (2) interfacial freezing at the liquid–liquid interface, modulated by the concentration of hexadecanol, which alters surface tension and promotes wettability transitions.

The physics of curvature switching arises from two interdependent mechanisms:

1. Interfacial freezing:
  Previous studies^35,37,38^ have shown that interfacial or surface freezing occurs at the interface between an aqueous surfactant solution (e.g., CTAC, CTAB) and a nonaqueous solution containing long-chain hydrocarbons (e.g., dodecane, hexadecane) with or without surfactants such as hexadecanol. This phenomenon arises as surfactants form a crystalline monolayer at a specific temperature, driven by enhanced lateral van der Waals interactions between hydrocarbon chains within the adsorbed film. During cooling, a critical temperature, *T*_s_, is reached at which a crystalline monolayer forms at the interface between the aqueous phase (DI water) and the nonaqueous phase (hexadecane, oil). This interfacial layer consists of molecules bound across the interface, including C_18_TAB, hexadecane (C_16_H_34_), and hexadecanol (CH_3_(CH_2_)_15_OH). The formation of this crystalline layer alters the surface tension at the liquid–liquid interface, typically decreasing it upon cooling and increasing it upon heating, in contrast to the typical thermally induced surface tension variations.^35^ The *T*_s_ can be tuned by varying the concentration of the cosurfactant (CH_3_(CH_2_)_15_OH), which modulates the temperature range of interfacial solidification.
2. Temperature-dependent adsorption and desorption of C_18_TAB:
  On the aqueous side, the adsorption and desorption of C_18_TAB surfactant molecules at the solid–liquid interface are strongly temperature-dependent. As temperature increases, more C_18_TAB molecules migrate from the bulk solution or the liquid–liquid interface to the wall surface (water–solid interface). This accumulation modifies the local wettability, transitioning the surface from hydrophilic to hydrophobic. C_18_TAB is a cationic surfactant composed of a hydrophilic quaternary ammonium head and a hydrophobic alkyl tail. The solid surface, which is generally negatively charged, electrostatically attracts the positively charged headgroups upon contact with water. The hydrophobic tails then orient outward, reducing the affinity for water molecules and effectively rendering the surface more hydrophobic.^21,35,37^ The migration of surfactants away from the liquid–liquid interface alters the interfacial tensions, which in turn adjusts the equilibrium contact angle to maintain consistency with Young’s equation
1 $$\sigma_{23}=\sigma_{13}+\sigma_{12}\cos\theta_{e}$$
  where σ_23_, σ_13_, and σ_12_ are the surface tensions at the oil-solid, water–solid, and water–oil interfaces, respectively, and θ_e_ is the equilibrium contact angle. During cooling, this process reverses: the adsorbed C_18_TAB molecules desorb from the wall, restoring the intrinsic hydrophilic nature of the solid glass substrate.

The coupled effects of thermally driven adsorption/desorption and interfacial freezing govern the observed curvature-switching behavior. Their interplay leads to dynamic regulation of capillary pressure and interfacial shape, enabling active control over wetting states that would otherwise be static in conventional systems. According to the Young–Laplace equation, the capillary pressure depends on both the curvature and the interfacial tension (eq 2). Therefore, variations in either curvature or surface tension at the interface lead to changes in capillary pressure, which in turn result in changes in wettability or displacement of the meniscus due to the induced pressure imbalance.

2 $$P_{c}=\sigma_{12}\left(\frac{1}{R_{1}}+\frac{1}{R_{2}}\right)$$

Here *P*_c_ is the capillary pressure, σ_12_ is the surface tension at the liquid–liquid interface, and *R*_1_ and *R*_2_ are the principal radii of curvature (Figure 2).

**Figure 2:**
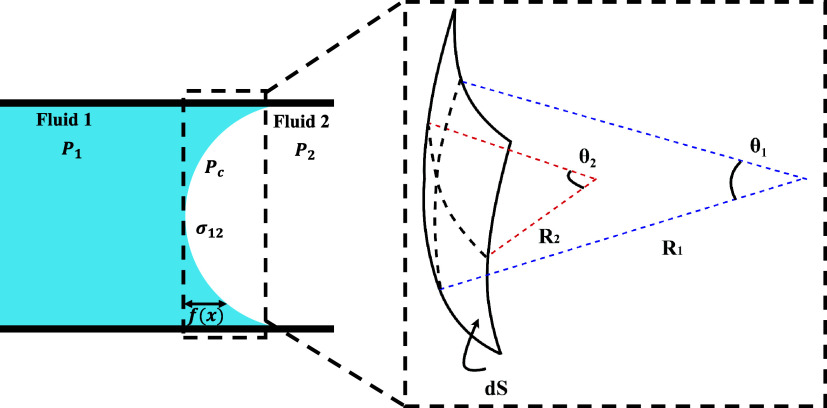
Schematic representation of the principal radii of curvature for a meniscus inside a capillary.

## Experimental Design

Building on the theoretical framework of curvature switching, this section describes the experimental and computational methodologies designed to quantify the coupled effects of temperature-dependent surfactant adsorption/desorption and interfacial freezing on meniscus curvature, wettability, and capillary pressure. The experimental design directly reflects the governing mechanisms outlined in the theory by combining (i) well-controlled aqueous–oil surfactant systems to tune interfacial tension and solid–liquid wettability, (ii) precise thermal regulation to trigger adsorption, desorption, and interfacial crystallization, and (iii) high-resolution optical imaging to capture dynamic meniscus evolution. Capillary geometries are selected to probe curvature effects, while surface tension measurements provide independent characterization of thermally driven interfacial changes. Dynamic surface tension measurements were performed using a maximum bubble pressure tensiometer to quantify the temperature-dependent interfacial properties of surfactant solutions. Additional details regarding the experimental setup, temperature control, and measurement procedure are provided in Supporting Information. Automated image-processing algorithms are then employed to extract contact angle and displacement data in a time-resolved manner, enabling direct comparison with Young–Laplace relations. Together, the hardware, software, and analysis protocols form an integrated experimental framework that translates the theoretical mechanisms of curvature switching into measurable, reproducible observations.

### Preparation of Materials and Capillary Assembly

This study investigates temperature-dependent interfacial phenomena in a variety of liquid–liquid and liquid–air systems, with a focus on interfacial freezing, wettability modulation, and active curvature control induced by surfactant systems. All experiments utilized ultrapure deionized water (18.2 MΩ cm) as the aqueous phase. The cationic surfactant octadecyltrimethylammonium bromide (C_18_TAB, Sigma-Aldrich, 98% purity) was dissolved in water at 1 mM as the baseline solution, well above its critical micelle concentration (CMC ≈ 0.25 mM), to ensure micelle formation and maximum interfacial activity.^39,40^ The aqueous solutions were stirred at 50 °C for over 40 min to guarantee complete dissolution.

The nonaqueous phase includes hexadecane (C_16_H_34_, TCI, >98% purity) used as a model system. To induce interfacial crystallization at elevated temperatures, hexadecane was doped with trace amounts of a long-chain fatty alcohol (hexadecanol, CH_3_(CH_2_)_15_OH, Aldrich, 99% purity, <25.6 mM), chosen for its ability to coassemble with surfactants at the interface and promote solidification over a broad temperature range.^41,42^ The oil–alcohol mixture was preheated and stirred above the melting point of the alcohol (typically >60 °C) to ensure homogeneity.

To explore geometric effects on interfacial dynamics, the fluids were confined within borosilicate glass capillaries (VitroCom) of various cross-sectional geometries, including rectangular (1 mm × 0.1 mm, 2 mm × 0.1 mm) and circular (0.5 mm inner diameter) capillaries. Each capillary was partially filled with the nonaqueous phase via capillary action. The same end of the capillary was then immersed in the aqueous surfactant solution, enabling spontaneous capillary-driven imbibition and positioning the interface at the center of the capillary. This approach facilitated direct visualization of the interface under controlled conditions. Figure 3 illustrates the experimental setup, including the hot plate with magnetic stirring used to prepare surfactant solutions, the capillary loading procedure, and the geometries of the capillaries employed in the experiments.

**Figure 3:**
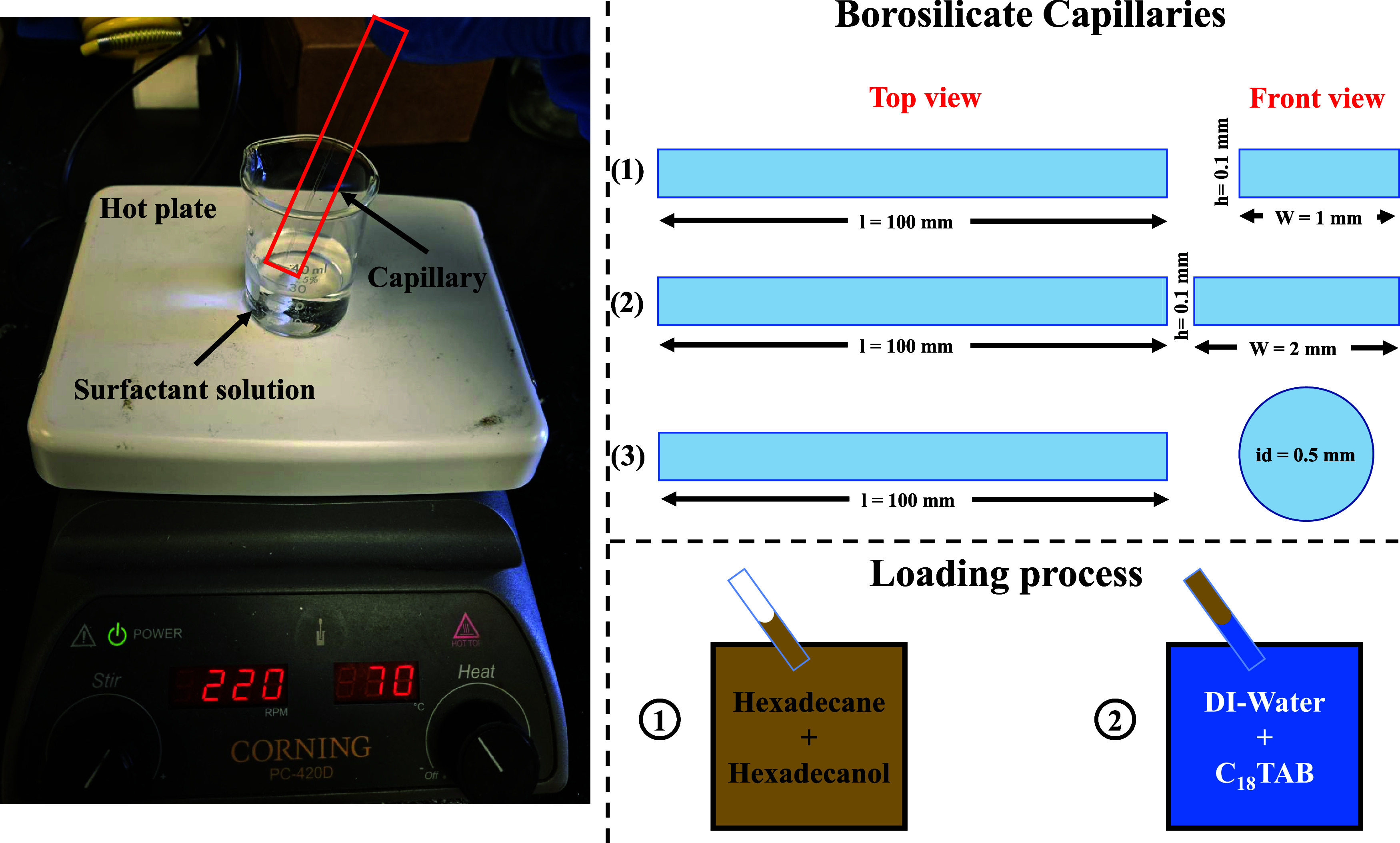
Experimental setup showing the preparation of surfactant solutions on a hot plate with magnetic stirring, and the capillary loading process via immersion into the aqueous solution. The figure also shows the three capillary geometries used: two rectangular (1 × 0.1 mm^2^ and 2 × 0.1 mm^2^) and one circular (0.5 mm inner diameter).

To avoid premature interfacial freezing during loading, both oil and aqueous solutions were maintained at temperatures ≥50 °C, significantly above the known interfacial freezing temperature (∼38 °C for 19.2 mM hexadecanol).^37^ This thermal protocol ensured the system was initialized in the liquid–liquid interfacial state, enabling controlled heating/cooling to induce recrystallization. It is important to note that reversing the order of loading yielded qualitatively identical results, confirming the robustness and symmetry of the interfacial phenomena under investigation.

### Thermal Control and Optical Imaging

After loading the capillary with the prepared solutions, the glass capillary was placed (wide face down) onto a thermo-electric cooler (TEC), which was used to control the temperature at the interface under constant current (CC) mode. As shown in Figure 4, this assembly was then horizontally mounted on a custom-built heat sink along with a cooling (water) reservoir and positioned on a manual XYZ translation stage. The water bath was filled with ambient-temperature water or ice water, depending on the desired direction of heat flow, to maintain the TEC’s efficiency and sustain a stable temperature during both cooling and heating. The temperature control setup utilized a single-stage Peltier element (Thorlabs) for bidirectional thermal regulation by switching the polarity of the electrical leads. The temperature at the target surface of the TEC was measured using a K-type thermocouple (relative error of <0.04%), and recorded using a National Instruments (NI) data acquisition system (DAQ NI-9210) using LabVIEW (LabVIEW 2025 Q1). This configuration enabled precise temperature control across the 20–50 °C range, with a resolution of 0.1 °C. Controlled scanning rates were employed to systematically assess the influence of thermal history on the interfacial phenomena. As depicted in the actual experimental setup shown in Figure 4, the surface of the TEC was covered with a high-temperature thermal tape. This thermally conductive layer served a dual purpose: it promoted uniform heat transfer across the contact surface while simultaneously allowing adequate background contrast for imaging.

**Figure 4:**
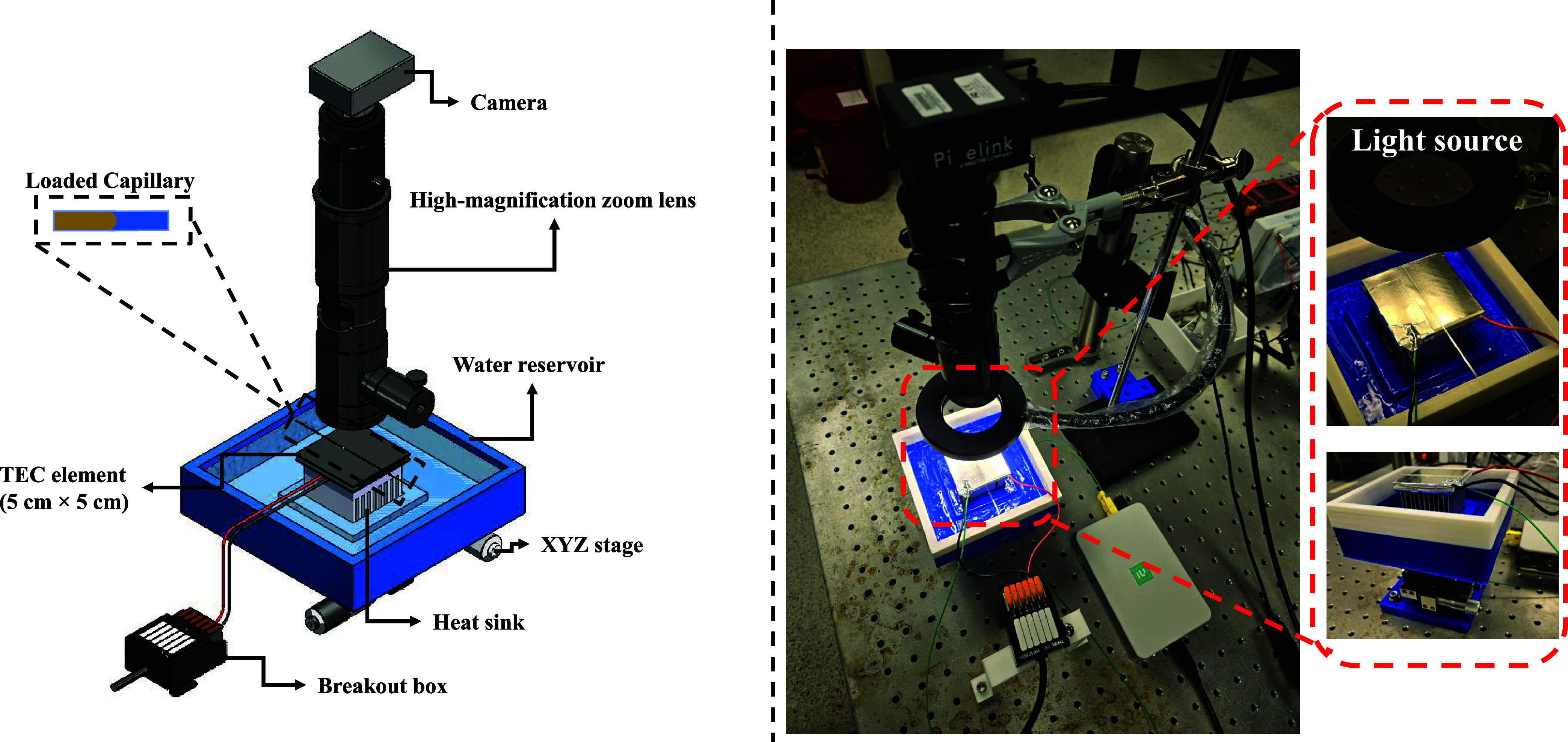
Schematic diagram and actual pictures of the experimental setup. The entire setup was supported on a manual XYZ translation stage for precise positioning.

High-resolution imaging was conducted using a high-magnification zoom lens system (Thorlabs MVL12 × 3Z), coupled with a CMOS color camera (Pixelink PL-D775CU-T) for real-time video acquisition. All imaging was performed with long working distance optics, thereby minimizing spurious thermal gradients that could influence interfacial dynamics. Bright-field illumination was provided by horizontally aligned ring-shaped LEDs. This optical setup enabled high spatial and thermal fidelity in visualizing and analyzing phenomena such as interfacial freezing, wettability transitions, meniscus curvature modulation, and dynamic motion, under tightly regulated environmental conditions.

### Analysis

#### Image Processing: Contact Angle and Meniscus Motion Trackers

To investigate interfacial dynamics and evaluate the capability for active control over the shape and position of the interface, three main parameters were measured: contact angle (θ), displacement (*d*), and surface tension (σ). Optical techniques are used to measure the contact angle and meniscus displacement inside the capillary tubes. Image processing was applied to video frames to quantify the contact angle and displacement during both heating and cooling under various conditions. While many studies^43^ have used ImageJ for contact angle measurements, this approach is prone to human error in manual line selection, which can introduce inconsistencies in the data. Moreover, frame-by-frame contact angle or displacement measurements in ImageJ are not straightforward, as manual measurements are required for each frame of a video, which typically contains more than 1000 frames. Hence, an automated program was required to process all frames in each video during heating or cooling to extract θ and *d* for every frame.

In this study, an automated contact angle measurement algorithm, contact angle tracking (CAT), is developed to quantify the dynamic behavior of a meniscus from a recorded video sequence. The process begins by loading the video and the corresponding temperature data. A region of interest (ROI) (Figure 5) is defined around the meniscus (the three-phase contact region) to focus the analysis and exclude extraneous areas. This cropping ensures that subsequent edge detection and contact angle extraction steps operate only on relevant pixels. For each video frame, the ROI is extracted and filtered using a bilateral filter to reduce noise while preserving interface information. A constant threshold is then applied to produce a binary image that highlights the meniscus edges. The Canny edge detection method is then applied, with edge components smaller than 1000 pixels removed to reduce noise for further analysis. Density-Based Spatial Clustering of Applications with Noise (DBSCAN)^44^ was employed to cluster edge pixels into spatially coherent clusters, based on point density. This provided a robust method for separating meaningful structures from noisy output of the edge detection algorithm. On average, each cluster contained more than 20 pixels, and each was fit with a first-order (linear) least-squares model. This provided the relative angles of these features with respect to the frame’s horizontal. The appropriate cluster corresponding to the meniscus was extracted post hoc, based on the cluster exhibiting the most significant temporal and physical consistency. This was a relatively straightforward determination, as the other clusters corresponded to the walls of the capillary or the line on the tape in the background, which did not vary over time and remained consistent across experiments. Due to the length of the capillary being parallel to the frame, the relative angles extracted corresponded to the appropriate contact angle. Frames with insufficient or invalid data are skipped, ensuring that only meaningful measurements contribute to the final data set.

**Figure 5:**
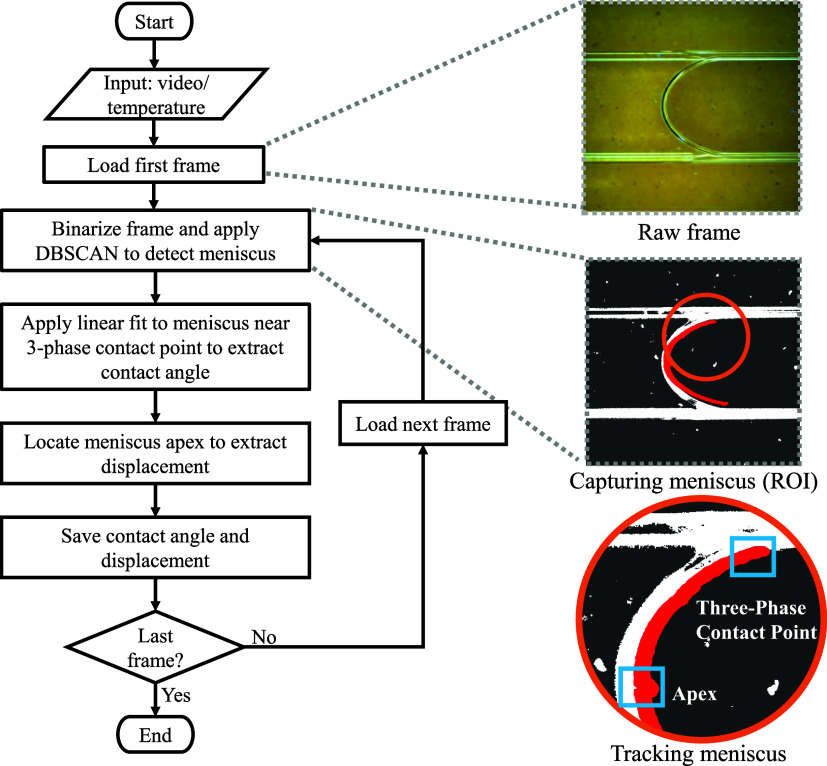
Schematic workflow for CAT and displacement. The process includes loading video and temperature data, cropping the meniscus region, filtering and thresholding, edge detection, clustering edge points, linear fitting to extract the meniscus slope, and tracking the contact angle and displacement.

As part of the contact angle tracking algorithm, a dedicated procedure was implemented to track the meniscus center (apex), in which the preprocessed image was binarized, and the corresponding point was tracked across successive frames. The meniscus displacement (*d*) is defined as the position of the meniscus apex tracked over time relative to its location in the first frame of the experiment. The apex was identified from the binarized images and automatically tracked across successive frames using the CAT algorithm. Figure 5 illustrates the workflow for CAT and displacement measurement, providing a robust, automated approach for quantifying dynamic wetting phenomena and facilitating correlation with thermal effects and other experimental parameters.

To evaluate the accuracy of the CAT method, a Young–Laplace based fit was used to match the theoretical shape of the meniscus. This fit was derived from the Young–Laplace eq (eq 2), based on the observed meniscus profile in the capillary. The Young–Laplace equation relates the pressures of the two phases to the capillary pressure

3 $$P_{1}-P_{2}=\sigma_{12}\kappa$$

where *P*_1_ and *P*_2_ are the pressures of the wetting and nonwetting phases, respectively, and κ is the curvature of the meniscus. A method to numerically solve the meniscus shape in right circular cylinders was developed by Concus^45^ and is modified for planar geometries here. Nondimensionalizing using a characteristic pressure scale σ_12_/*L*

4 $$\frac{P_{1}-P_{2}}{\sigma_{12}/L}=L\kappa$$

let, $\lambda =\frac{P_{1}-P_{2}}{\sigma_{12}/L}$

5 $$\lambda =\frac{f^{\prime \prime }}{\sqrt{1+f^{\prime 2}}}$$

where *f*(*x*) is the nondimensional height of the meniscus (Figure 2). The resulting differential equation

6 $$f^{\prime \prime }-\lambda \sqrt{1+f^{\prime 2}}=0$$

is solved numerically using an Adams-Bashforth-Moulton method to satisfy the following boundary conditions

$$\begin{array}{ll} f^{\prime }=0 & \mathrm{at}\;x=0\\ f^{\prime }=\tan\left(\frac{\pi }{2}-\theta \right) & \mathrm{at}\;x=1 \end{array}$$ 7

A shooting method similar to Concus^45^ is used to find the value of λ that satisfies the second boundary condition. An adaptive threshold edge detection^46^ is first used to identify points along the meniscus. An optimization method is used to find θ that minimizes the *R* ^2^ value between the numerical solution and the image data.

As shown in Figure 6, contact angles measured using three methods, ImageJ, CAT, and the Young–Laplace (YL) fit, are compared. The comparison between ImageJ and CAT shows that at temperatures of 25, 30, 35, 40, and 45 °C, the measured values are nearly identical, within ±2°. However, ImageJ-based measurements are manual and labor-intensive; therefore, they were performed for only five frames corresponding to these temperatures. As such, this comparison alone does not fully validate the CAT results. To further verify CAT, the YL fit was employed. Since the YL fit is based on an equilibrium assumption, it cannot accurately capture dynamic behavior. During the switching process, temperature-driven surfactant redistribution results in surface-tension gradients along the interface. These gradients cause the meniscus curvature to deviate from equilibrium solutions, such as those by Concus.^45^ Consequently, CAT is compared with the YL fit only before and after the switching process, where good agreement within 3° is observed. These results demonstrate that CAT is a reliable and dynamic tool for predicting the contact angle throughout the process; therefore, CAT is used for all subsequent contact angle measurements.

**Figure 6:**
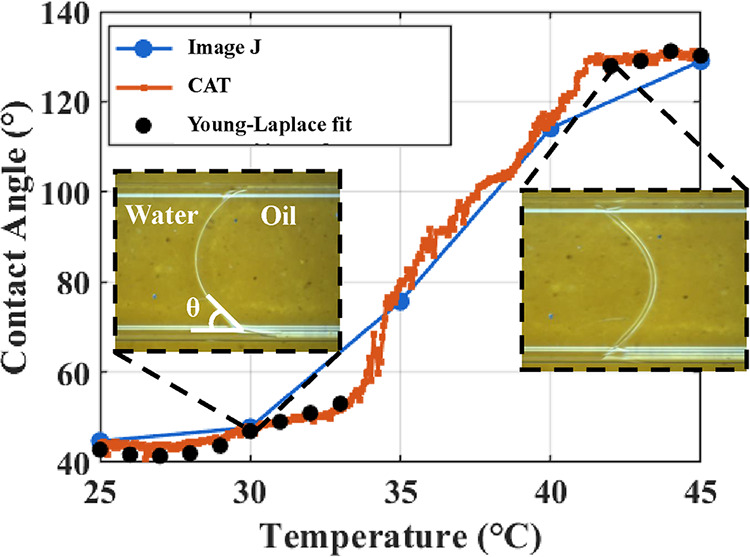
Comparison of contact angle measurements obtained using ImageJ, CAT, and the Young–Laplace fit at various temperatures. CAT provides dynamic measurements throughout the process, while ImageJ was limited to selected frames. The agreement with the Young–Laplace fit confirms the accuracy and reliability of CAT.

#### Data Acquisition and Uncertainty Analysis

Each experiment was repeated three to five times under identical conditions. Temperature was measured with a K-type thermocouple in direct contact with the top surface of the thermoelectric cooler (TEC), beneath the capillary. The temperature measurement is calibrated against an infrared (IR) thermometer, which itself was precalibrated using a NIST-traceable reference thermometer. This two-step calibration established a precise voltage–temperature correlation curve, enabling temperature measurements with an estimated uncertainty of ±0.1 °C.

The temperature and optical data were synchronized through timestamp alignment between the LabVIEW logs and video acquisition system, ensuring that thermal and geometrical changes were correlated within ±0.1 s. This approach minimized temporal misalignment and ensured consistency across all thermal–optical data sets. During experiments, the supplied current was manually adjusted to maintain the target steady-state temperature, achieving equilibrium based on real-time feedback displayed in LabVIEW. The uncertainty associated with repeatability, arising from small variations between nominally identical runs, is incorporated into the reported results.

## Results and Discussion

In this section, the experimental results are discussed. The effects of meniscus displacement, curvature, and surface tension on the active interfacial dynamics are examined under heating and cooling, with and without surfactants. The roles of surfactant concentration, thermal gradients, capillary geometry, and contact angle hysteresis are also discussed to highlight mechanisms that can control interfacial dynamics.

### Effect of Temperature-Sensitive Surfactants

This section examines the influence of temperature-sensitive surfactants on interfacial behavior and wetting dynamics, focusing on meniscus displacement and contact angle variations across liquid–air and liquid–liquid interfaces. Both pure liquids (DI water, hexadecane) and surfactant-modified systems (DI water + C_18_TAB, hexadecane + hexadecanol (CH_3_(CH_2_)_15_OH), and their combinations) are investigated. Complementary surface tension measurements relate interfacial property changes to the observed wetting behavior.

Experiments first consider the simpler, surfactant-free systems to establish the governing principles of capillary pressure and interfacial balance based on established theory (eqs 1–2). The baseline cases include DI water–air, hexadecane-air, and DI water-hexadecane systems, with each case repeated three to five times under identical conditions. Results are summarized in Figure 7, where panels (a)–(c) show meniscus displacement (*d*, mm), contact angle (θ, °), and surface tension (σ, mN/m) as functions of temperature (°C), respectively. For all systems in the 1 mm-wide capillary, temperature was increased from 25 to 45 °C (red solid) and then decreased back to 25 °C (blue dotted). As shown in Figure 7(a), the water–air meniscus, indicated by the circular marker, shifts toward the water side by approximately 1 mm during both heating and cooling, with a standard deviation within ±0.2 mm, primarily due to evaporation. In contrast, contact angle changes remain below 5° (Figure 7(b)), while interfacial tension decreases by about 4 mN/m with temperature (Figure 7(c)), indicating that the dynamics are mainly driven by evaporation and surface tension variations rather than intrinsic wettability changes.

**Figure 7:**
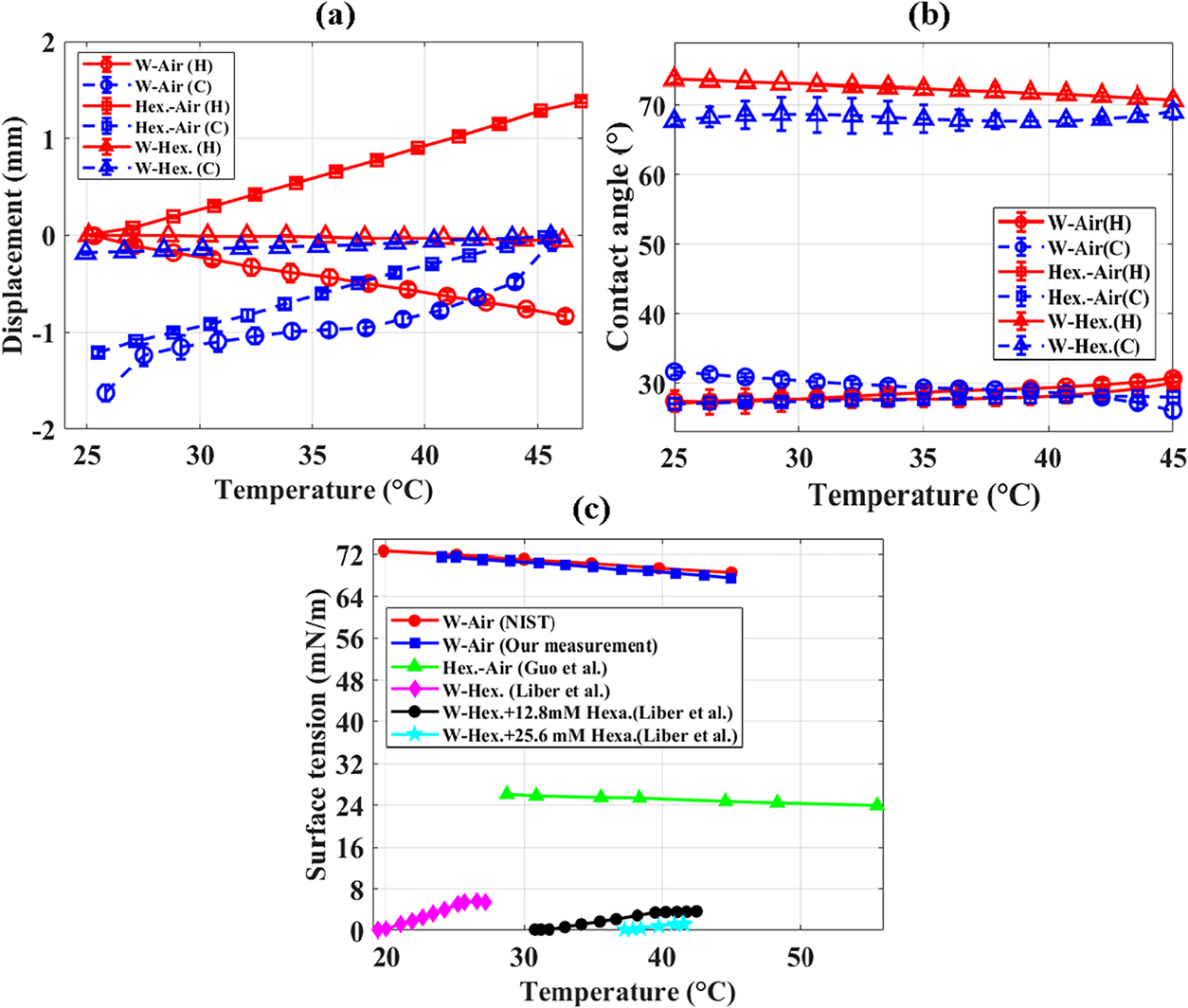
Meniscus and interfacial properties of pure fluid systems as a function of temperature between 25 and 45 °C. (a) Meniscus displacement, (b) contact angle, and (c) surface tension.^37,47^

The hexadecane (oil)-air interface, depicted by the square marker (Figure 7(a)), shows the opposite trend: the meniscus moves toward the oil side (positive displacement) upon heating and retracts during cooling. Since hexadecane evaporation is negligible due to its high boiling point (286 °C), displacement is driven mainly by temperature-dependent surface tension (Figure 7(c)). A decrease in σ during heating increases pressure on the oil side, producing a positive displacement per the Young–Laplace equation, while the reverse occurs during cooling. However, contact angle variations remain minimal (<5°).

For the water-hexadecane interface, indicated by the triangular marker, the meniscus displacement is about an order of magnitude smaller than in liquid–air systems, with a maximum of 0.17 mm during cooling. This minor shift reflects the balance between pressure and interfacial tension variations at the liquid–liquid interface. As shown in Figure 7(c), the interfacial tension at the water-hexadecane interface is significantly lower than that of the water–air interface, with values of approximately 5.6 and 71 mN/m at 27 °C, respectively, based on data reported by Liber et al.^37^ Consequently, θ increased notably, from ∼30° to 72° on the hydrophilic borosilicate surface, to compensate for the change in surface tension, emphasizing the distinct role of interfacial tension variations compared to air interfaces. The corresponding temperature-dependent oil–water interfacial tension for different hexadecanol concentrations is shown in Figure 7.^37^ This effect of surface tension on wettability and meniscus displacement will be further manipulated later by adding surfactants to actively control the interfacial dynamics.

The influence of temperature-sensitive surfactants was examined by adding C_18_TAB to water and hexadecanol (CH_3_(CH_2_)_15_OH) to hexadecane. Results for meniscus displacement and contact angle are shown in Figure 8. Panels (a) and (b) (top) present the displacement as a function of temperature for liquid–air (left) and liquid–liquid (right) interfaces, respectively, while panels (c) and (d) (bottom) show the corresponding contact angle variations.

**Figure 8:**
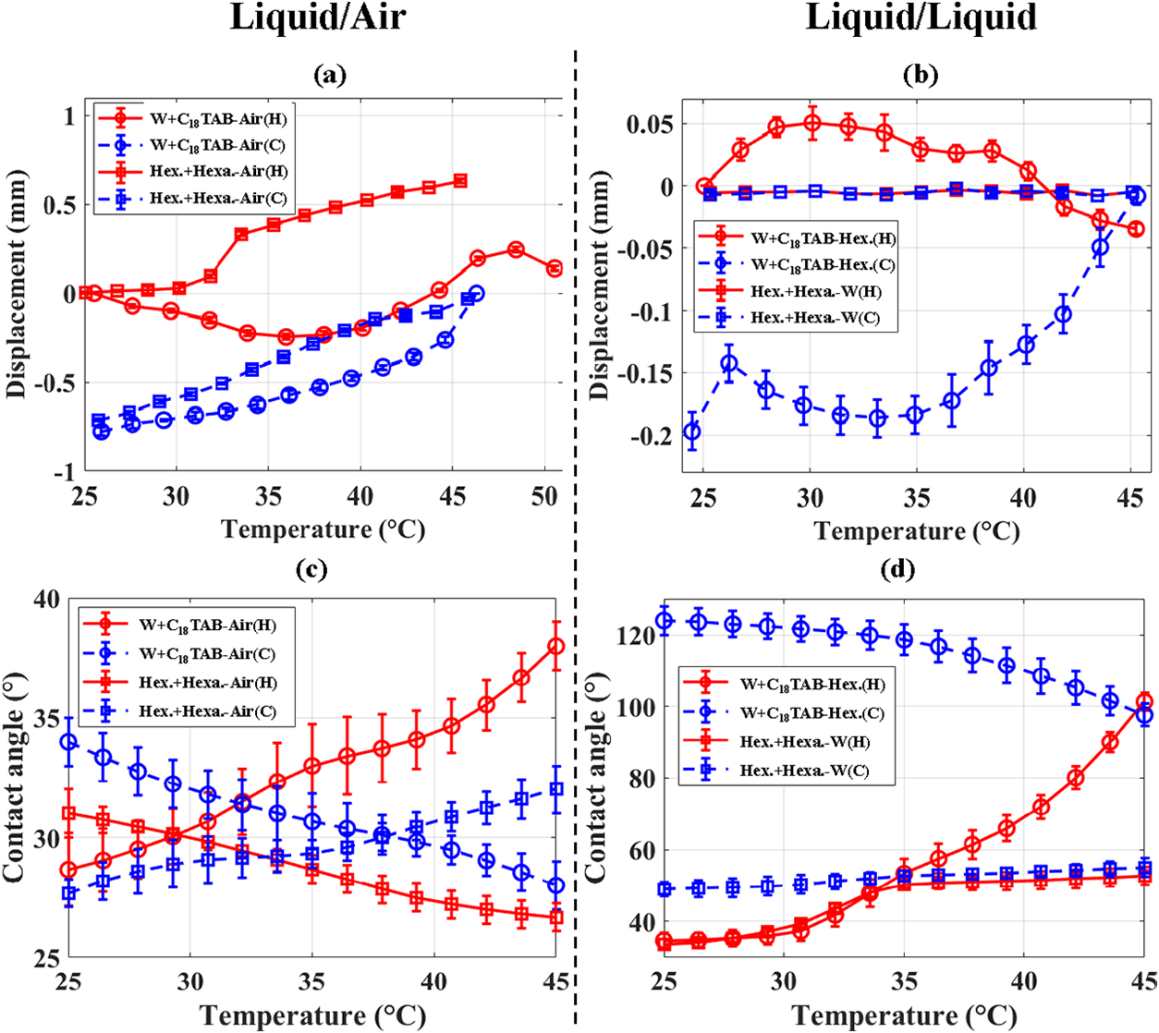
Meniscus displacement (a, b), and contact angle (c, d) for air interfaces (DI-water + C_18_TAB-air, hexadecane + hexadecanol­(Hexa.)-air) and liquid–liquid interfaces (DI-water + C_18_TAB-hexadecane, and hexadecane + hexadecanol­(Hexa.)-DI-water). Heating curves are shown in red and cooling curves in blue.

At the water + C_18_TAB-air interface, depicted by the circular marker, the meniscus displacement is lower than the case without surfactants but it exhibits greater temperature sensitivity. This effect is particularly pronounced during heating, when the displacement tends to either slow or reverse direction. This is due to C_18_TAB adsorption onto the wall, rendering the surface more hydrophobic and modifying the liquid–solid and liquid–air interfacial tensions. Such tunable behavior is advantageous for microfluidic applications requiring active control of displacement direction. The presence of the surfactant alters the temperature dependence of interfacial tension, enhancing meniscus tunability, with displacement variations remaining within ±0.1 mm across repeated trials. In the hexadecane + CH_3_(CH_2_)_15_OH-air system (indicated by the square marker), the displacement is lower than in the pure hexadecane-air interface, reflecting reduced interfacial energy and increased thermal sensitivity. As shown in Figure 8(b), displacement in liquid–liquid systems is generally smaller than in liquid–air interfaces. In the water + C_18_TAB-hexadecane system, displacement remains minimal but is still thermally modulated, while the addition of hexadecanol further suppresses motion by influencing the interfacial freezing range.^35^

Figure 8(c),(d) show strong temperature-dependent contact angle variations resulting from nonequilibrium adsorption/desorption of C_18_TAB at the liquid–air and solid–liquid interfaces. During heating (Figure 8(c)), C_18_TAB partially detaches from the water–air interface and adsorbs onto the negatively charged glass wall, transiently increasing wall hydrophobicity and contact angle. Complete curvature switching is inhibited by electrostatic attraction between the cationic surfactant headgroups and the negatively charged water–air interface. When the temperature reaches 45 °C and is briefly held, partial relaxation toward equilibrium occurs as surfactant molecules return to the liquid–air interface, lowering the contact angle relative to the heating at 45 °C. Additional differences between heating and cooling at 45 °C arise from transient evaporation and condensate formation, which increase vapor pressure on the air side and further reduce the contact angle. Upon cooling, C_18_TAB molecules are perturbed by the temperature variation and partially migrate from the liquid–air interface to the solid–liquid interface, leading to an increase in the contact angle. Figure 8(d) demonstrates that curvature switching occurs during heating even without hexadecanol, confirming that surfactant redistribution alone can induce nonequilibrium wetting transitions. In contrast, switching does not occur during cooling because desorption from the wall is hindered by the weaker affinity between C_18_TAB and hexadecane compared to its strong electrostatic attraction to borosilicate during heating.

To further illustrate the temperature-dependent kinetics of C_18_TAB molecules, surface tension measurements were performed for the water + C_18_TAB-air interface in a borosilicate beaker, mimicking the interfacial dynamics observed in the capillary. The effects of temperature and concentration on surface tension were examined, as shown in Figure 9(a–c). Panel (a) shows the variation of surface tension with bubble lifetime at a concentration of 4 mM. An equilibrium value is obtained after a characteristic time. The dynamic response is temperature dependent. Panels (b) and (c) depict the effects of temperature and concentration on the equilibrium surface tension (values obtained after stabilization at a given temperature or concentration). Equilibrium surface tension decreases with increasing surfactant concentration, even beyond the CMC of C_18_TAB (0.25 mM). Moreover, the reduction in equilibrium surface tension with increasing temperature becomes more pronounced at higher concentrations (e.g., 4 mM), indicating enhanced interfacial activity of the surfactant at elevated temperatures. This behavior is attributed to temperature-dependent adsorption and desorption kinetics at the glass wall.

**Figure 9:**
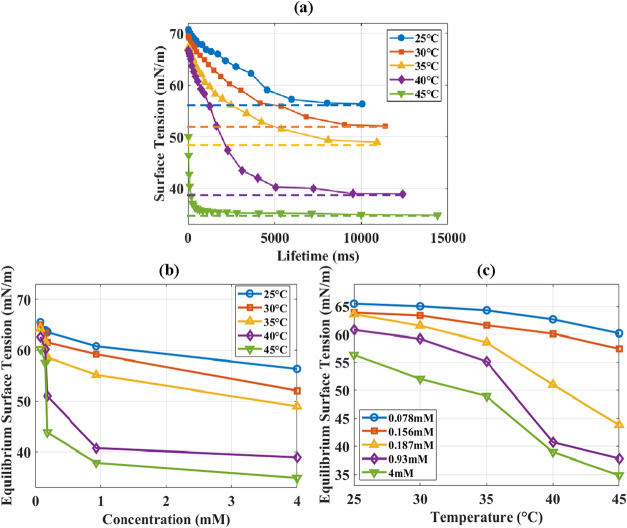
Surface tension of the water + C_18_TAB-air interface showing the effects of temperature, concentration, and bubble lifetime. (a) Dynamic surface tension at 4 mM C_18_TAB approaching equilibrium at different temperatures. (b) Equilibrium surface tension versus temperature for various concentrations. (c) Equilibrium surface tension versus C_18_TAB concentration at selected temperatures. Surface tension decreases with increasing concentration and shows stronger temperature dependence at higher concentrations, reflecting adsorption–desorption kinetics.

Finally, the combination of C_18_TAB in water and CH_3_(CH_2_)_15_OH in hexadecane exhibits significant contact angle variation and moderate meniscus displacement. Figure 10 shows meniscus snapshots between 25 and 45 °C during heating and cooling. During heating, the meniscus moves by approximately 0.5 mm, and the contact angle increases from 38° to 132°. During cooling, displacement is minimal (0.2 mm), while the contact angle decreases from 142° to 42°, confirming a reversible curvature-switching process. This is unexpected, as borosilicate glass is inherently hydrophilic. This behavior is attributed to the adsorption of C_18_TAB molecules from the aqueous phase or the liquid–liquid interface onto the glass surface, thereby reversing the native wettability.

**Figure 10:**
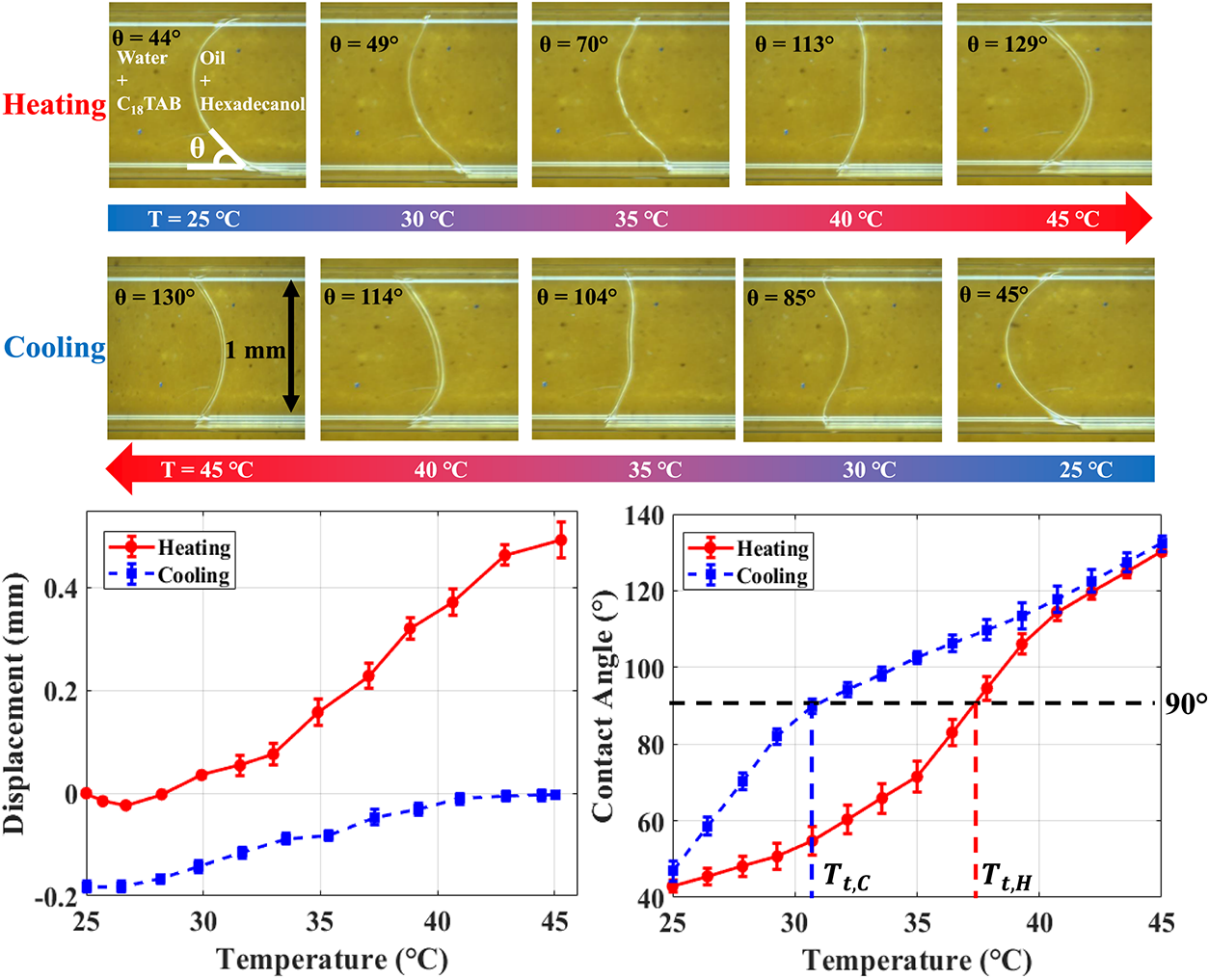
Snapshots of the DI-water + C_18_TAB-hexadecane + hexadecanol meniscus at different temperatures during heating and cooling, illustrating meniscus displacement and contact angle variations that demonstrate curvature switching.

Interestingly, the contact angle data in Figure 10 indicate that the temperature at which the contact angle reaches 90° is approximately 37 °C during heating and 30 °C during cooling. This suggests that curvature switching exhibits a unique thermal contact angle hysteresis that depends on the direction of heat transfer, governed primarily by the surfactant adsorption–desorption kinetics. Videos of the curvature-switch experiments during both heating and cooling are provided in the Supporting Information.

### Effect of Surfactant Concentration

This section examines the influence of the concentration of temperature-sensitive surfactants on the contact angle and the transition temperature (*T*_t_). Here, *T*_t_ is defined as the temperature at which the contact angle reaches 90°, marking the point of curvature switching. A consistent difference in *T*_t_ is observed between heating and cooling cycles, arising from two coupled phenomena: the temperature-dependent adsorption and desorption of surfactant molecules and interfacial freezing at the liquid–liquid interface.

To systematically study these effects, the concentrations of C_18_TAB in water and CH_3_(CH_2_)_15_OH in hexadecane are varied. As shown in Figure 11, the first series of experiments (left column) fixes the CH_3_(CH_2_)_15_OH concentration in oil at 19.2 mM while varying C_18_TAB in water at 0.5, 1, and 2 mM. In the second series (right column), the C_18_TAB concentration is held at 1 mM, and the CH_3_(CH_2_)_15_OH concentration is adjusted to 9.6, 19.2, and 38.4 mM. This systematic approach enables us to isolate how surfactant concentration influences the onset of curvature switching and the modulation of meniscus wettability.

**Figure 11:**
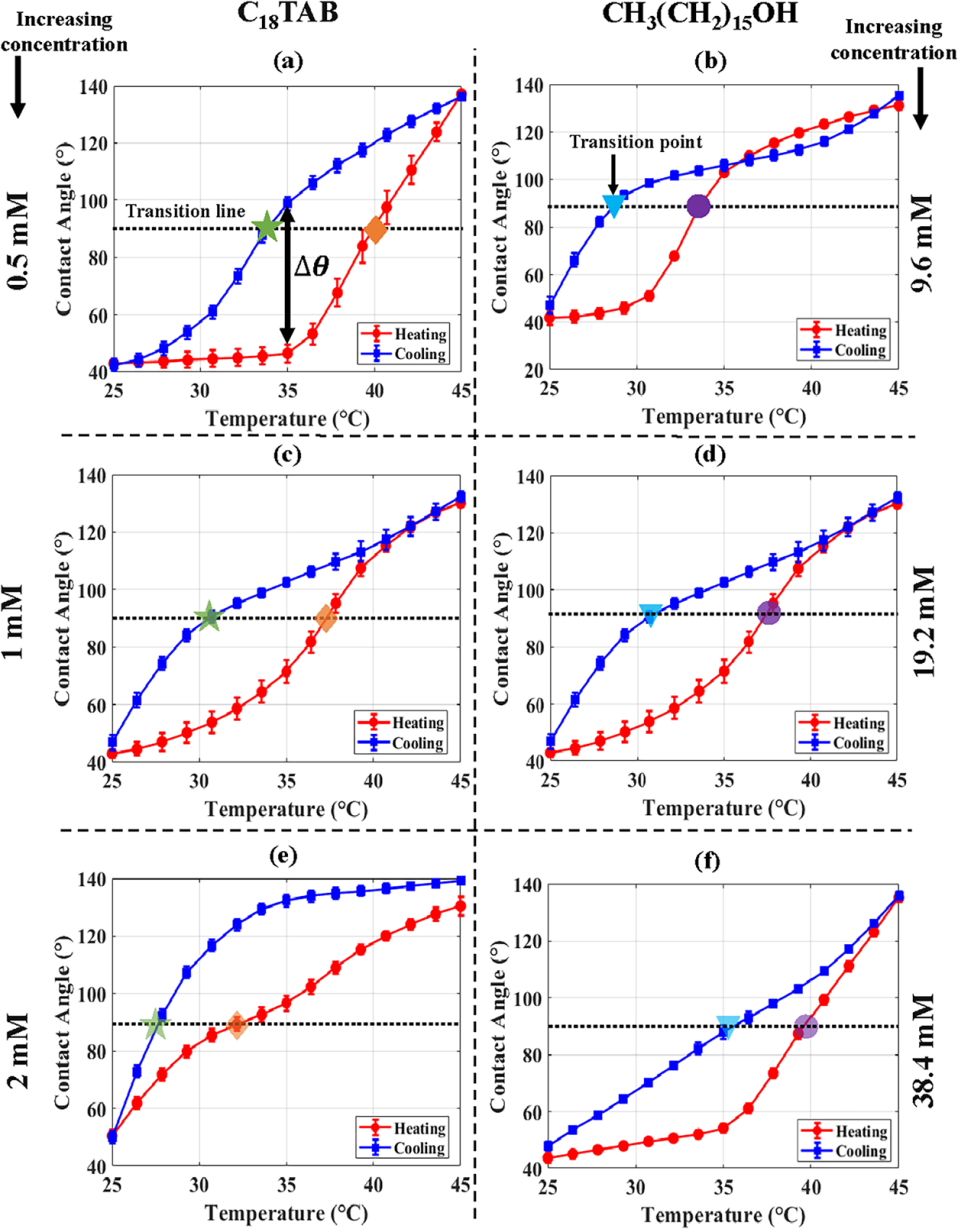
Contact angle as a function of temperature for different surfactant concentrations. Panels (a, c, e) show the effect of varying C_18_TAB concentration in water (0.5, 1, and 2 mM, respectively) while the hexadecanol concentration in hexadecane is fixed at 19.2 mM. Panels (b, d, f) show the effect of varying hexadecanol concentration in hexadecane (9.6, 19.2, and 38.4 mM, respectively) while the C_18_TAB concentration in water is fixed at 1 mM. Red and blue solid curves correspond to heating and cooling cycles, respectively. Increasing C_18_TAB lowers the transition temperature, whereas increasing hexadecanol raises it, highlighting the distinct roles of aqueous- and oil-phase surfactants in curvature switching.

Figure 11(a,c,e) show the contact angle as a function of temperature for different C_18_TAB concentrations. As the C_18_TAB concentration increases, the curvature-switching behavior shifts to lower temperatures, reflected in variations in the hysteresis envelope and in the difference Δθ between cooling and heating curves at the same temperature. Similarly, Figure 11(b,d,f) illustrate the effect of varying hexadecanol concentration in hexadecane (with C_18_TAB fixed at 1 mM). The resulting changes in switching behavior differ from those observed in the C_18_TAB-variation cases. As hexadecanol concentration increases, the curvature switching occurs at higher temperatures. This indicates that the adsorption/desorption of C_18_TAB and interfacial freezing each influence curvature switching in distinct ways. Collectively, these trends demonstrate that both mechanisms can be tuned independently to control wettability and transition temperature.

Figure 12(a),(b) illustrate the dependence of *T*_t_ (as indicated by the markers in Figure 11(b),(c)) on C_18_TAB and hexadecanol concentrations. Increasing C_18_TAB lowers *T*_t_, whereas increasing hexadecanol raises *T*_t_, highlighting the contrasting roles of aqueous versus oil-phase surfactants in controlling curvature switching. During heating, increasing C_18_TAB concentration from 0.5 to 2 mM enables premature switching, consequently the transition temperature, *T*_t_, is reduced from approximately 40 to 33 °C, indicating that higher surfactant availability requires lower temperature for curvature switching. This trend is true for both heating and cooling and can be explained by temperature-dependent molecular kinetics. At elevated concentrations, adsorption to the wall becomes active at 34 °C, leading to sufficient adsorption of surfactant molecules that hydrophobize the wall and trigger switching. However, during cooling, since switching occurs at a lower temperature than during heating, as illustrated in Figure 10, molecules become less active at lower temperatures (e.g., 27–34 °C), which affects their desorption.

**Figure 12:**
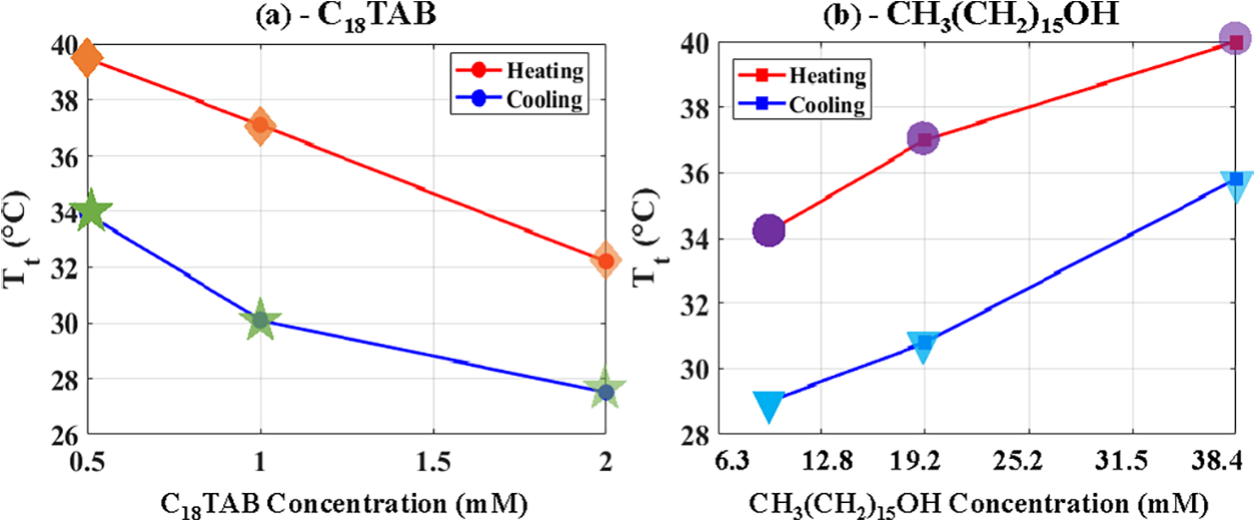
Dependence of transition temperature *T*_t_ on surfactant concentration. (a) Variation of *T*_t_ with C_18_TAB concentration in water for heating and cooling cycles, with hexadecanol fixed at 19.2 mM. (b) Variation of *T*_t_ with hexadecanol concentration in hexadecane for heating and cooling cycles, with C_18_TAB fixed at 1 mM. Increasing C_18_TAB lowers *T*_t_, whereas increasing hexadecanol raises *T*_t_.

Figure 12(b) indicates that the trend is reversed compared to the results in the aqueous phase. Increasing hexadecanol concentration raises *T*_t_ during both heating and cooling. This behavior is primarily attributed to interfacial freezing. As a long-chain fatty alcohol, hexadecanol increases the freezing point of hydrocarbons. With higher concentrations, interfacial freezing occurs at elevated temperatures.^35^ The curvature switching is therefore closely linked to the interfacial freezing temperature, which can be tuned by hexadecanol concentration. It is also noteworthy that thermal contact angle hysteresis Δθ between heating and cooling persists in all cases, reflecting the asymmetric kinetics of adsorption and desorption at the interfaces, as discussed in more detail in a subsequent section. Overall, these results demonstrate that surfactant concentration provides a powerful tool for actively tuning contact angle, transition temperature, and consequently capillary pressure. The ability to manipulate *T*_t_ through molecular design and concentration control has significant implications for microfluidics, thermal management, and other applications requiring precise interfacial control.

### Effect of Capillary Geometry

After examining the influence of temperature-sensitive surfactant concentration on wettability and interfacial dynamics, we next investigated the role of capillary geometry. Temperature-driven curvature changes of fluid menisci were studied in rectangular and circular borosilicate glass capillaries filled with surfactant solutions (water + C_18_TAB and oil + hexadecanol). Three geometries were tested: rectangular capillaries of 1 mm and 2 mm width, and circular capillaries of 0.5 mm diameter (Figure 3). The corresponding meniscus displacement and contact angle data are presented in Figure 13 for both heating and cooling. Figure 13(a) illustrates that the largest displacements (up to ∼2.5 mm) occurred in the widest capillary (2 mm rectangular), driven by curvature switching as confirmed by the slope of the displacement curve. The larger displacement observed in wider capillaries arises because the meniscus radius and interfacial area increase with capillary size. This lowers the capillary pressure that must be overcome for the meniscus motion. As a result, the same curvature switching event produces a larger axial shift of the interface in larger capillaries compared to narrower ones.

**Figure 13:**
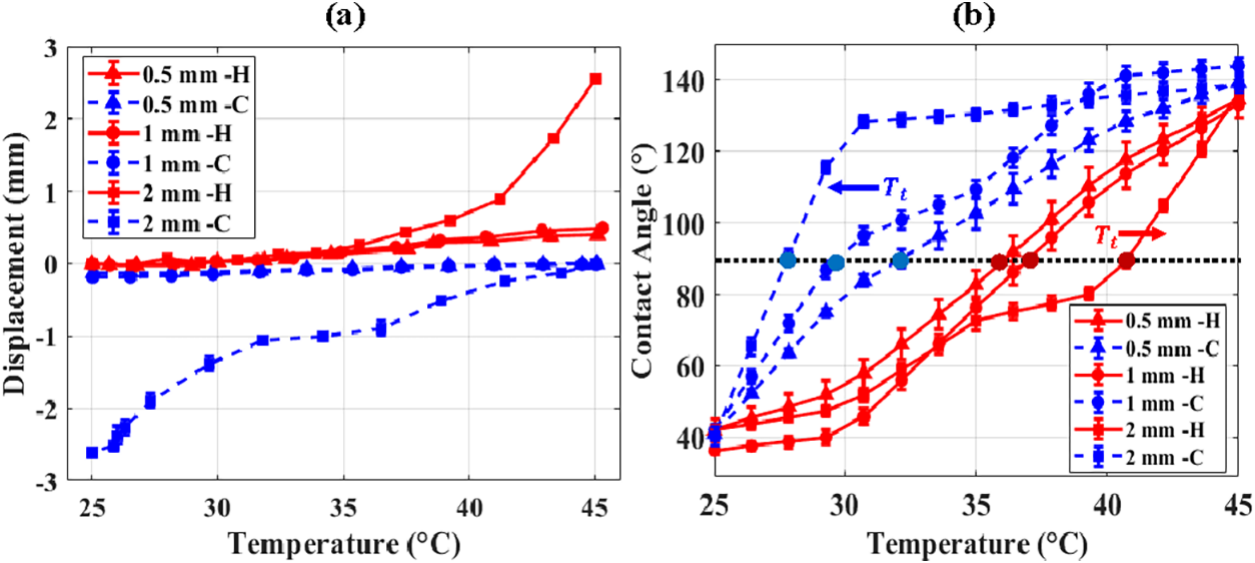
Effect of capillary geometry on meniscus dynamics. (a) Meniscus displacement and (b) contact angle as a function of temperature in rectangular capillaries (1 mm and 2 mm width) and circular capillaries (0.5 mm diameter) during heating and cooling cycles. Corresponding transition temperature *T*_t_ for each geometry, illustrating geometry-dependent hysteresis between heating and cooling.

Figure 13(b) shows that the transition temperature, *T*_t_ (black line), varied with geometry and heating/cooling. As capillary size is increased, *T*_t_ increased on heating and reduced on cooling. This demonstrates a progressively widening hysteresis envelope as the capillary size increases. The widening of the hysteresis envelope with increasing capillary size could be attributed to an increase in C_18_TAB adsorption area and the corresponding heat of adsorption. The higher adsorption of C_18_TAB in the larger sized capillaries generates appreciable intrinsic heat and is the likely cause of the increase in the transition temperature in the heating cycle. Conversely, transition temperature decreases during the cooling cycle due to C_18_TAB desorption and interfacial freezing. These opposing shifts increase the separation between the heating and cooling transition temperatures as the geometric length scale increases. These results demonstrate a temperature-tunable, reversible, and reproducible method for controlling meniscus curvature via surfactant-mediated wettability switching. The interplay between adsorption and desorption enables precise thermal control over interfacial geometry, with repeatability and hysteresis confirmed over multiple cycles.

### Thermal Contact Angle Hysteresis

A consistent trend across all experiments is the presence of thermal hysteresis in the contact angle, defined as the difference between cooling and heating values at the same temperature (Δθ_CH_ = θ_c_ – θ_h_). Figure 14(a),(b) show this behavior for the averaged data sets of C_18_TAB and CH_3_(CH_2_)_15_OH, respectively. In most cases, the contact angle during cooling exceeds that during heating, yielding positive hysteresis; the only exception is the lowest hexadecanol concentration (9.6 mM), where a small negative value is observed. As shown in Figure 14(a), different C_18_TAB concentrations exhibit similar trends, with 1 and 2 mM showing peaks near 30–35 °C. Similarly, as illustrated in Figure 14(b), hexadecanol displays maximum hysteresis within the same temperature range as C_18_TAB. However, deviations at the lowest concentrations of both surfactants arise from near-CMC conditions (CMC: 0.25 mM for C_18_TAB and 6.4 mM for CH_3_(CH_2_)_15_OH), where limited bulk surfactant leads to slower or incomplete adsorption during heating. During cooling, desorption occurs more readily due to lower interfacial coverage, weakening or reversing the typical positive hysteresis.

**Figure 14:**
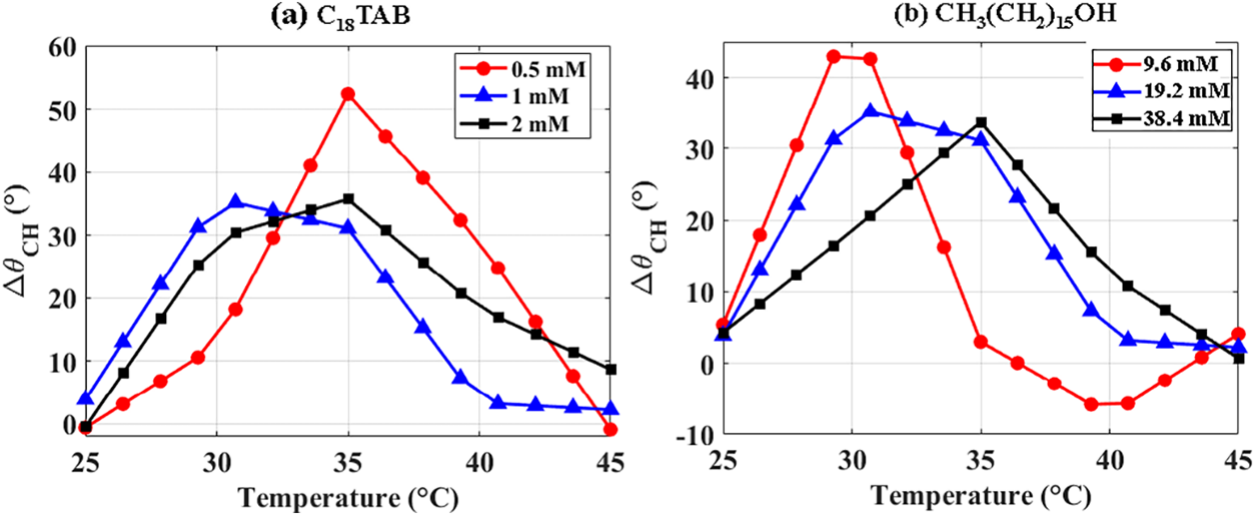
Contact angle hysteresis (Δθ_CH_) for different surfactant concentrations. (a) Hysteresis for aqueous C_18_TAB at concentrations of 0.5, 1, and 2 mM. (b) Hysteresis for hexadecanol in hexadecane at concentrations of 9.6, 19.2, and 38.4 mM. Positive values indicate larger contact angles during cooling compared with heating.

Across all systems, *T*_t_ during heating is consistently higher than during cooling, with offsets of 6–10 °C depending on geometry, surfactant type, and concentration. These results confirm that hysteresis originates from asymmetric adsorption/desorption kinetics, further enhanced by interfacial freezing. Two key conclusions follow: (i) thermal hysteresis in contact angle is inherent to temperature-responsive surfactant systems, and (ii) surfactant concentration relative to the CMC strongly modulates hysteresis, with near-CMC conditions producing qualitatively different behavior from supra-CMC regimes. This distinction provides a useful design parameter for applications that require tunable or controlled wetting hysteresis.

Another notable feature is that hysteresis envelopes consistently originate and terminate near zero, suggesting that mechanical hysteresis due to surface roughness or pinning is minimal. Instead, hysteresis increases with temperature, peaks near the curvature switching point, and decreases once switching is complete. This pattern indicates that hysteresis is closely linked to the molecular processes driving the transition. To further verify that the observed hysteresis was not caused by mechanical pinning or surface roughness effects, experiments were repeated using multiple borosilicate capillary tubes with identical dimensions, and consistent results were obtained across all capillaries. Practically, hysteresis presents both challenges and opportunities. For microfluidic and thermal management applications, it must be considered when precise reversibility of meniscus position or capillary pressure is required. Conversely, hysteresis can be exploited to design functional behaviors such as bistability, thermal memory, or diode-like responses, in which the fluid interface retains information about the last thermal cycle. Thermally induced contact angle hysteresis can thus serve as a valuable design parameter for engineering advanced interfacial systems.

### Effect of Temporal Temperature Gradient

In addition to surfactant concentration and capillary geometry, the temperature ramp rate was varied to assess its effect on interfacial dynamics. Heating and cooling processes with different ramp rates were applied to the water + C_18_TAB - hexadecane + CH_3_(CH_2_)_15_OH system. Thermal ramps ranging from 0.01 °C/s to 2 °C/s were imposed while monitoring meniscus curvature and contact angle. Figure 15 presents the contact angle measurements and transition temperatures under these varying ramp rates. Panel (a) shows the contact angle as a function of temperature for slow and fast heating/cooling, while panel (b) summarizes the corresponding transition temperatures. Remarkably, the results indicate that neither the magnitude of the contact angle nor the *T*_t_ is significantly affected by the temporal gradient. Both slow and fast ramps produce overlapping curves for heating and cooling, and *T*_t_ remains essentially constant across the range of applied rates.

**Figure 15:**
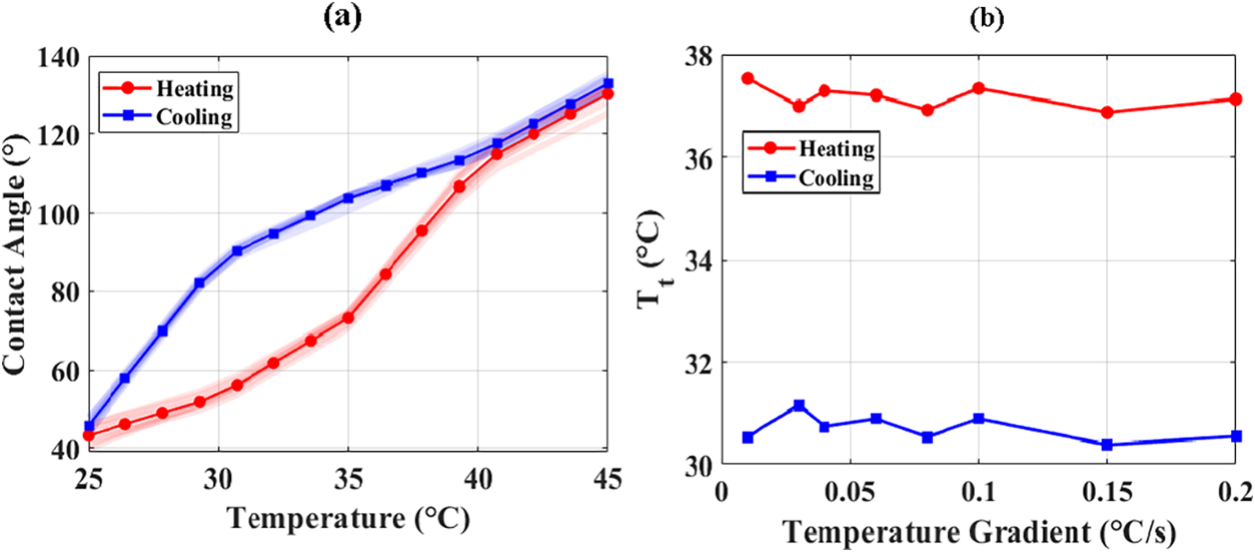
Effect of temporal temperature gradient on curvature switching. (a) Averaged contact angle as a function of temperature for different heating and cooling ramp rates (0.01–2 °C/s). All runs are depicted using translucent lines and clearly match in magnitude and trend. (b) Corresponding transition temperature *T*_t_ as a function of ramp rate, showing negligible dependence on the applied temperature gradient.

This observation suggests that the curvature switching process is primarily governed by temperature and is independent of the heat transfer rate. In other words, the system equilibrates rapidly enough that the interfacial composition and structure respond quasi-statically to temperature variations. Within the tested range, temporal temperature gradients do not introduce measurable lag in meniscus response or alter the hysteresis behavior. This indicates that temperature-driven control of meniscus curvature and contact angle is robust across a wide range of thermal ramp rates. In addition, we repeated heating and cooling cycles (over 15 cycles) and held the system at a constant temperature for at least 10 min prior to switching, confirming that the meniscus curvature switching is fully reversible and stable, with no observable degradation in surfactant performance or interfacial shape. For applications in microfluidics, adaptive optics, or thermal management, precise control over temperature ramp speed is therefore less critical, simplifying thermal actuation protocols while still achieving reproducible curvature switching and interfacial control.

## Conclusions

This study systematically explored the dynamic behavior of temperature-sensitive surfactants in confined capillary systems, highlighting the mechanisms governing curvature switching, meniscus motion, and wettability modulation. Through a combination of controlled heating/cooling protocols, high-resolution imaging, and continuous contact angle tracking, several key insights were obtained:

- Surfactant concentration strongly governs curvature switching: increasing C_18_TAB in water lowers the transition temperature, *T*_t_, while increasing hexadecanol in hexadecane raises *T*_t_, highlighting the contrasting roles of aqueous and oil-phase surfactants in tuning interfacial properties.
- Capillary geometry modulates meniscus displacement and hysteresis: wider capillaries show larger displacements, whereas narrower capillaries exhibit smaller, more gradual meniscus motion.
- Thermal contact angle hysteresis is intrinsic and reproducible across all surfactant systems. Contact angles during cooling consistently exceed those during heating, resulting in positive hysteresis. This behavior is linked to interfacial freezing and molecular kinetics rather than experimental variability or surface roughness. Thermal hysteresis in contact angles arises from asymmetric adsorption/desorption kinetics, with offsets increasing with increasing geometry.
- Temporal temperature gradients have minimal impact: within the tested heating and cooling rates, neither the contact angle magnitude nor the transition temperature *T*_*t*_ was significantly affected by varying temporal temperature gradients. This indicates that curvature switching is dominated by temperature-dependent interfacial kinetics rather than the speed of thermal actuation, simplifying practical implementation.
- Combined effects enable active, reversible, and tunable interfacial control: by carefully selecting surfactant concentration, capillary geometry, and thermal protocols, predictable curvature switching, controllable meniscus displacement, and programmable wettability transitions can be achieved.

Collectively, these findings provide a comprehensive understanding of the factors governing surfactant-mediated interfacial dynamics in micro- and macro-confined systems. Beyond the fundamental insights, the demonstrated ability to thermally tune contact angle, curvature switching, and meniscus displacement offers practical opportunities for active control of liquid interfaces. Such temperature-responsive interfacial behavior can be exploited in applications including microfluidic flow regulation, adaptive optical components, and thermal management systems where reversible manipulation of fluid interfaces is desirable. The ability to control transition temperature through surfactant concentration and capillary geometry further provides a versatile design parameter for engineering responsive capillary-driven devices. Future work will focus on extending these concepts to more complex geometries, multiphase transport environments, and integrated microfluidic platforms to enable practical implementations of thermally actuated interfacial systems.

## Supplementary Material





## Data Availability

Data will be made available on request.

## Notes

Additional details of the surface tension measurements, including the experimental setup, temperature control, and measurement procedure using the maximum bubble pressure tensiometer (PDF)
Videos of curvature switching during heating (25–45 °C) and cooling (45–25 °C) (ZIP)

## Nomenclature

**Abbreviations**
LCST: lower critical solution temperature
CAT: contact angle tracking
CMC: critical micelle concentration
YL: Young–Laplace
**Chemical Species**
C_18_TAB: octadecyltrimethylammonium bromide
CH_3_(CH_2_)_15_OH: hexadecanol
C_16_H_34_: hexadecane
**Symbols**
κ: curvature
Δθ_CH_: thermal contact angle hysteresis
*f*(*x*): nondimensional height
*P*_c_: capillary pressure
*R*_1_ & *R*_2_: principal radii
*L*: characteristic length scale
σ_23_: surface tension at the oil-solid interface
σ_13_: surface tension at the water–solid interface
σ_12_: surface tension at the water–oil interface
*T*_s_: interfacial freezing critical temperature
λ: nondimensional pressure
θ_e_: equilibrium contact angle
*x*: nondimensional length
