# Supplementary material for: An Experimental Study of Interfacial Dynamics Control Using Temperature-Sensitive Surfactants
Source: Langmuir. 2026 Apr 28;42(20):14045–59. doi: 10.1021/acs.langmuir.5c06748 (PMC13217622; doi:10.1021/acs.langmuir.5c06748)
Supplement: Supplementary file 1 [file la5c06748_si_001.pdf]

Supporting Information

An Experimental Study of Interfacial Dynamics Control Using Temperature-Sensitive Surfactants

Amirhosein Sarchami, Saaras Pakanati, Ayaaz Yasin, Milind A. Jog, and Kishan Bellur*

Department of Mechanical and Materials Engineering
University of Cincinnati, Cincinnati, Ohio 45220, United States

*Email: bellurkn@ucmail.uc.edu

Table of Contents

1. Surface tension measurements, including the experimental setup, temperature control, and measurement procedure using the maximum bubble pressure tensiometer.

2. Curvature switching videos (CS_Heating.mp4, CS_Cooling.mp4) during heating (25–45 °C) and cooling (45–25 °C) demonstrating thermally induced meniscus curvature transitions.

1 Surface tension measurements

To investigate the effect of temperature-dependent adsorption and desorption kinetics of C_{18}TAB in the aqueous phase, dynamic surface tension measurements were performed using a maximum bubble pressure tensiometer (SINTERFACE BPA-2S). The measurements were conducted to quantify the dynamic surface tension of aqueous solutions containing different concentrations of C_{18}TAB at various temperatures (Figure 1). The measurements focused on the DI water–air interface for both pure DI water and DI water containing C_{18}TAB.

In the maximum bubble pressure method, gas bubbles are periodically generated at the tip of a submerged capillary connected to the tensiometer. The maximum pressure required to form each bubble corresponds to the capillary pressure at the bubble apex and is related to the surface tension through the Young–Laplace equation. In the present experiments, compressed air was used as the working gas for bubble generation. The pressure fluctuations during bubble formation were recorded by the tensiometer and converted into dynamic surface tension values.

The liquid samples were placed in a glass beaker integrated with the tensiometer system. The temperature of the sample was controlled within the range of 25–45 °C using a temperature controller (Thorlabs TC300B) connected to a flexible resistive heater wrapped around

the beaker. The temperature was monitored using a thermocouple placed in the liquid to ensure accurate thermal control during the measurements. The dynamic surface tension measurements were performed at constant, controlled temperatures for at least 10 minutes, ensuring that the measured surface tension values correspond to equilibrium conditions.

For each concentration and temperature condition, the measurements were repeated three times to ensure reproducibility. The reported values correspond to the average equilibrium surface tension obtained from these repeated measurements. This experimental approach enabled the characterization of temperature-dependent variations in surface tension induced by surfactant adsorption at the liquid–air interface.

These measurements provided key interfacial parameters necessary for interpreting the meniscus behavior and wetting dynamics observed in the capillary experiments discussed in the main manuscript.

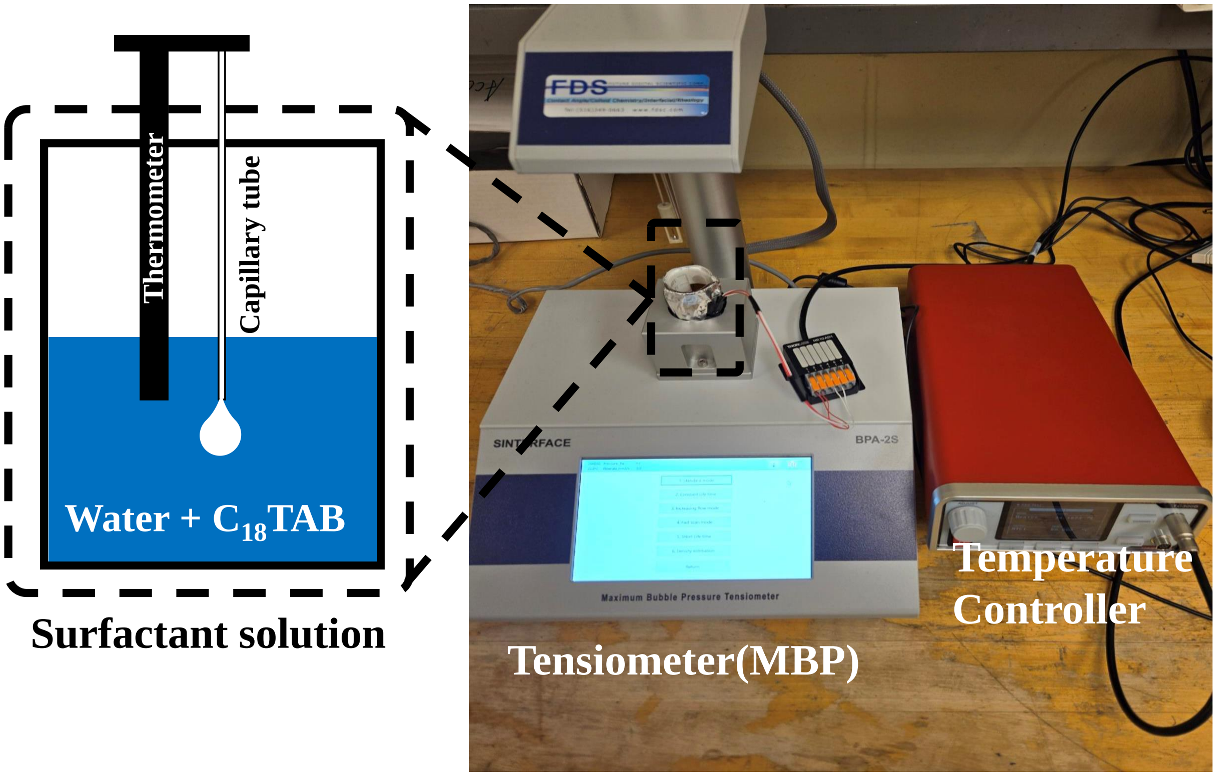

Figure 1: Schematic diagram and actual photograph of the surface tension measurement setup using the maximum bubble pressure tensiometer.

2 Curvature switching videos

1. **CS_Heating.mp4** – Curvature switching during heating from 25–45 °C (playback speed: 16×)

2. **CS_Cooling.mp4** – Curvature switching during cooling from 45–25 °C (playback speed: 16×)
